# Dual-Task Gait Analysis: Combined Cognitive–Motor Demands Most Severely Impact Walking Patterns and Joint Kinematics

**DOI:** 10.3390/life15071009

**Published:** 2025-06-25

**Authors:** Nenad Nedović, Slavica Mutavdžin Krneta, Stevan Jovanović, Danilo Vujičić, Žiga Kozinc, Dmitry Skvortsov

**Affiliations:** 1 Academy of Applied Studies Belgrade, College of Health Sciences, 11000 Belgrade, Serbia; nenad.nedovic@assb.edu.rs (N.N.); stevan.jovanovic@assb.edu.rs (S.J.);; 2 Faculty of Medicine, Institute of Medical Physiology, University of Belgrade, “Richard Burian”, 11000 Belgrade, Serbia; 3 Faculty of Health Sciences, University of Primorska, Polje 42, SI-6310 Izola, Slovenia; 4 Center for Brain and Neurotechnology, Moscow 117513, Russia; 5 Research and Clinical Centre, Moscow 107031, Russia

**Keywords:** spatiotemporal parameters, gait deterioration, aging population

## Abstract

Cognitive tasks significantly influence automated acts, such as walking. This study included 41 healthy individuals, who were over 65 years of age. We examined dual-task effects on the spatiotemporal and kinematic parameters of gait in older adults during four tasks carried out in single-task, cognitive, motor, and combined cognitive–motor conditions. An analysis of walking according to spatiotemporal and kinematic parameters was performed using an inertial movement analysis system. The combined task showed the most significant impairments, with substantially reduced gait speed (*p* < 0.001, r = −0.80), shorter stride length (*p* < 0.001, r = −0.82), and decreased hip flexion (*p* < 0.001, r = −0.80) compared to single-task walking. Cognitive tasks alone significantly affected gait speed (*p* = 0.001) and stride length (*p* = 0.001), while motor tasks showed minimal effects. The combined task also significantly increased double-support time (*p* < 0.001) and reduced single-support time (*p* = 0.001), indicating compensatory walking strategies. These findings demonstrate that concurrent cognitive–motor demands disproportionately impair gait, suggesting that clinical assessments should prioritize combined-task evaluation. The observed kinematic and spatiotemporal changes highlight the profound interdependence between cognitive function and automatic locomotor control during walking. It is likely that dual-task gait analysis may offer clinical utility for the early detection of cognitive–motor deficits.

## 1. Introduction

Assessing gait in older adults is a crucial component in evaluating their health status and fall risk, and it is particularly important in gerontology [1,2]. A gait cycle consists of a series of repeated steps that begin when one foot’s heel touches the ground and end when the same heel makes contact again [3].

Age-related walking decline involves factors like sensory (vision, proprioception, vestibular), neurological (sensorimotor integration), or motor (strength, balance) impairments. These lead to a slower walking speed and greater gait instability [4,5]. Cognitive functions also play a key role in walking, and age-related cognitive decline can further compromise gait stability and adaptability in older adults [6]. Research indicates that impairments in executive functioning and attention are correlated with increased variability in walking patterns, while memory has less influence on gait [7].

The dual-tasking methodology has proven to be an effective way to understand how cognitive tasks impact motor functions, including gait, in older adults [8]. This approach helps to reveal how effectively older adults can manage their balance and focus under dual demands and helps identify subtle gait impairments that may be missed during single-task assessments [9]. During evaluations, participants are given additional tasks while walking, such as arithmetic or verbal activities, to assess coordination and concentration [10,11,12]. Research has shown that difficulties in performing dual tasks may indicate underlying cognitive or physical impairments contributing to gait instability [13]; for instance, Hollman et al. [14] observed that older adults walk at a slower pace and exhibit increased stride variability during dual-task conditions compared to younger individuals, likely due to age-related declines in executive function and attention capacity, which are critical for managing dual-task demands.

Ever since their development, gait analysis technologies have evolved, with optoelectronic systems being highly accurate but costly and challenging to use daily in clinical settings [15,16]. Using reflective body markers and infrared cameras, these systems capture and quantify movement kinematics by analyzing the three-dimensional paths of the markers; however, marker-based motion analysis systems face limitations including skin motion artifacts, restricted natural movement due to cumbersome setups, occlusion issues, high costs, and limited real-world applicability [17]. In response to these limitations, inertial measurement units (IMUs) have gained interest as a more accessible and cost-effective alternative [18]. The use of IMU sensors enables precise movement tracking, provides valuable real-time data for gait analysis, and allows for the identification of subtle changes in gait patterns [19]. Based on prior evidence [20], we hypothesized that cognitive tasks would most significantly alter spatiotemporal and kinematic parameters compared to other tasks.

The current study aimed to analyze spatiotemporal and kinematic gait parameters using a system with IMU sensors for gait analysis in healthy older adult participants while they engaged in a challenging cognitive and motor dual task and to compare performance across task types.

## 2. Materials and Methods

### 2.1. Participants

This study included 41 healthy individuals who were over 65 years of age (Table 1). Participants with neurological disorders, orthopedic issues, diabetes mellitus, or cardiovascular conditions, which could affect gait and cognitive function, were also excluded. Those using mobility aids, like canes or walkers, were also excluded. All subjects were medication-free and reported a satisfactory quality of life and living conditions, and all had at least completed elementary school. Participants were recruited through personal networks and gave written informed consent before taking part in the study. The study was approved by the Ethical Committee of FSBI of the Center for Brain and Neurotechnology (No. 7 of 19 July 2021) and was performed in accordance with the ethical standards of the Declaration of Helsinki and its later amendments.

### 2.2. Experimental Protocol

Participants undertook 4 tasks: a basic self-paced walking task, a dual motor task, a dual cognitive task, and a combined motor and cognitive task [21]. The task order was randomized across participants. The dual motor task involved walking while holding a large, full glass of water without spilling it. The cognitive task involved performing serial “7” subtractions, starting from random numbers such as 100, 95, 90, or 105. The combined task required subjects to carry out both the glass-carrying and the subtraction exercises simultaneously, without prioritizing either task.

**Figure 1:**
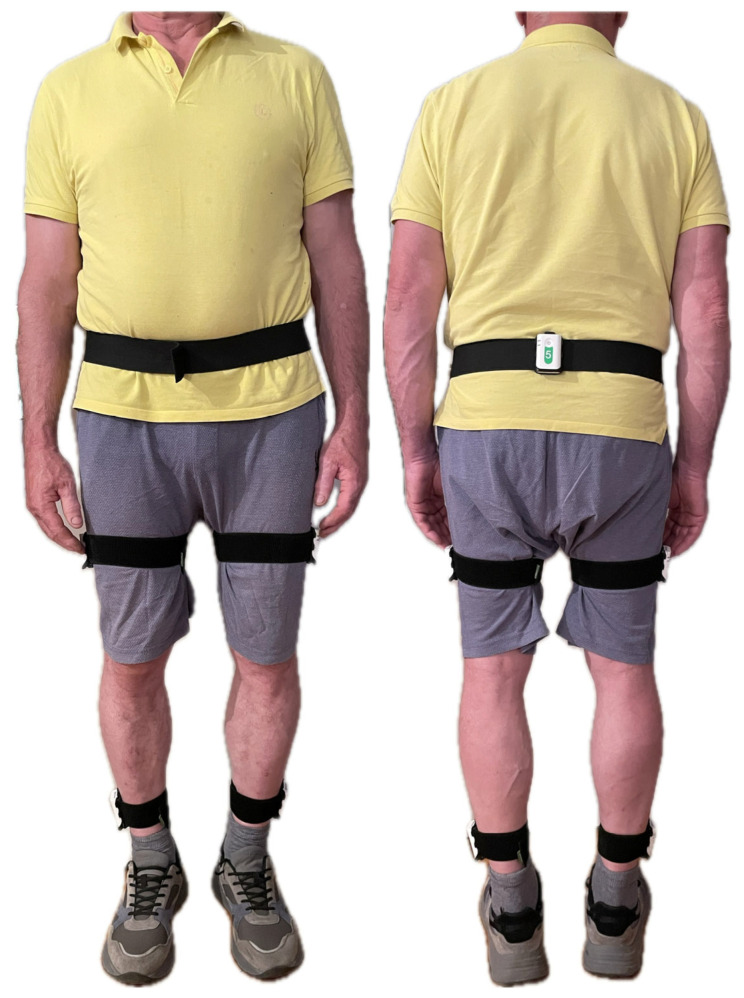
Setup of the measurement with IMU sensors.

Participants walked for 1 min down a 10 m corridor and back at a comfortable pace for each task “as if they were walking down the street”. Spatiotemporal and kinematic parameters of lower limb joints during walking were measured using the Steadys system with inertial sensors (Neurosoft, Ivanovo, Russia), which assessed movements in three dimensions [19]. Five inertial sensors were attached with cuffs to the lower back, thigh, and shank of both legs (Figure 1). Gait cycles were determined from the accelerometer data, and data were exported for each participant and task for further statistical analysis.

The spatiotemporal parameters were calculated by the software during 1 min of walking while performing the above-mentioned four tasks. The temporal parameters were gait cycle(s) and individual gait cycle phases, which were measured as a percentage of the gait cycle, namely the stance phase, single-support phase, and double-support phase. The spatial parameters were stride length (cm), gait speed (km/h), and foot clearance (cm)—the vertical height of the foot above the ground during the swing phase of the gait cycle, which was estimated using the appropriate algorithm in the Steadys system based on inertial sensor data. Kinematic parameters were also measured: lumbosacral (pelvis) maximum range of motion in three planes (flexion/extension, adduction/abduction, rotation amplitude), the hip joint’s maximum range of motion (flexion/extension, adduction/abduction, rotation amplitude), and the knee joint’s maximum flexion/extension amplitude.

### 2.3. Statistical Analysis

**Figure 2:**
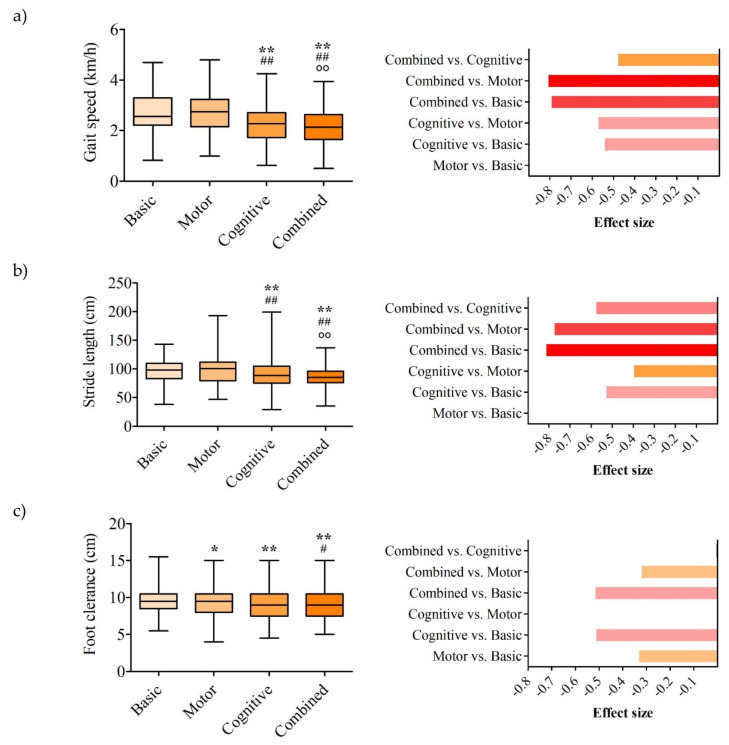
Results for (**a**) gait speed, (**b**) stride length, and (**c**) foot clearance. The left panel displays the median, interquartile range, minimum, and maximum values, along with statistically significant differences. The right panel shows the rank-biserial correlation coefficients for pairwise comparisons of parameters with statistically significant differences. * *p* < 0.05 vs. basic condition; # *p* < 0.05 vs. during the motor activity; ** *p* < 0.01 vs. basic condition; ## *p* < 0.01 vs. during the motor activity; ○○ *p* < 0.01 vs. during the cognitive activity.

**Figure 3:**
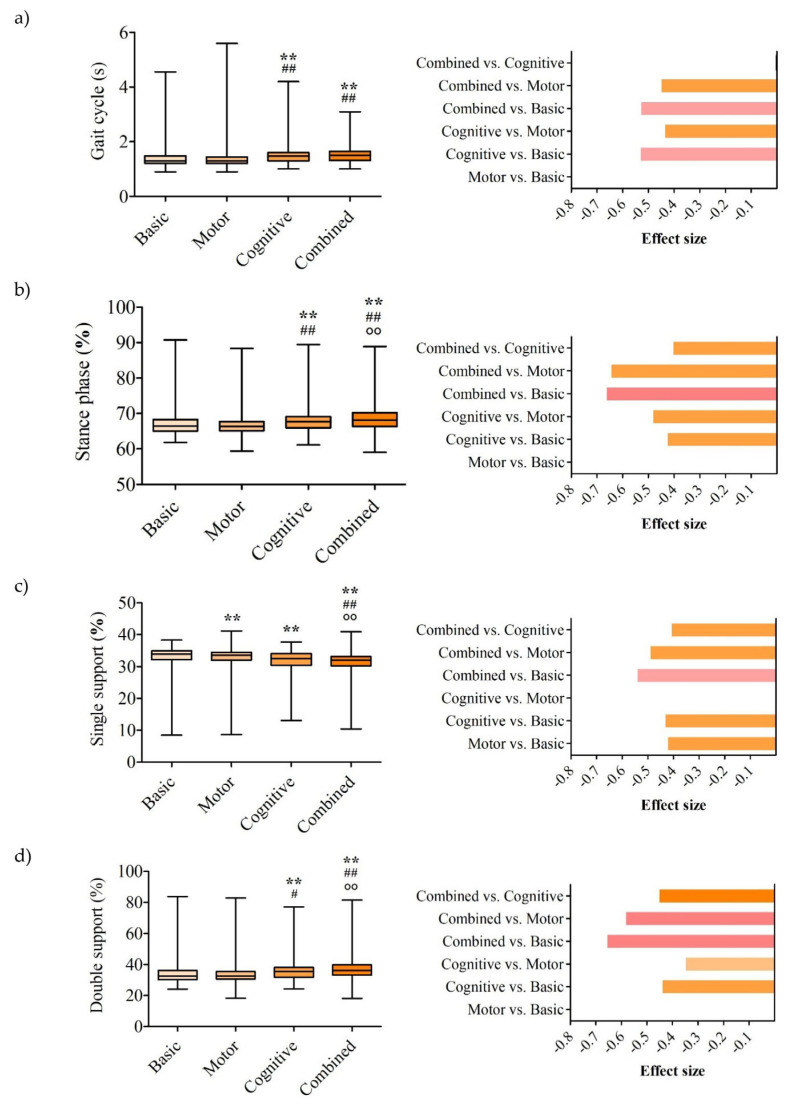
Results for (**a**) gait cycle, (**b**) stance phase, (**c**) single-support phase, and (**d**) double-support phase. The left panel displays the median, interquartile range, minimum, and maximum values, along with statistically significant differences. The right panel shows the rank-biserial correlation coefficients for pairwise comparisons of parameters with statistically significant differences. # *p* < 0.05 vs. during the motor activity; ** *p* < 0.01 vs. basic condition; ## *p* < 0.01 vs. during the motor activity; ○○ *p* < 0.01 vs. during the cognitive activity.

Statistical analysis was conducted using SPSS 17.0 (SPSS Inc., Chicago, IL, USA). Differences in gait parameters across four experimental conditions—basic, motor, mental, and combined—were analyzed using the Friedman test, followed by post hoc pairwise comparisons using the Wilcoxon signed-rank test since the data did not follow a normal distribution. Effect sizes were calculated using Kendall’s W for overall comparisons and rank-biserial correlation (r) for pairwise analyses. The spatiotemporal parameter results are presented in Figure 2, while kinematic parameters are presented in Figure 3. The left panel displays the median, interquartile range, minimum, and maximum values, along with statistically significant differences. The right panel shows the rank-biserial correlation coefficients (r) for pairwise comparisons of parameters with statistically significant differences.

## 3. Results

Table 1 shows the descriptive characteristics of the participants.

### 3.1. Spatiotemporal Parameters

Gait speed differed significantly across conditions (*p* < 0.001, W = 0.385; Figure 2a). Compared to basic conditions (2.60 [0.83–4.69] km/h), speed was significantly lower during the cognitive (2.30 [0.62–4.25] km/h, *p* = 0.001, r = −0.545) and combined tasks (2.13 [0.50–3.94] km/h, *p* < 0.001, r = −0.796), both with large effect sizes. The combined task also showed a significantly lower speed than the motor task (2.81 [0.99–4.80] km/h, *p* < 0.001, r = −0.811), with a large effect size, and the cognitive task (*p* = 0.003, r = −0.481), with a moderate effect size. No significant differences were observed between the motor and basic conditions (*p* = 0.455).

Stride length differed significantly across conditions (*p* < 0.001, W = 0.434; Figure 2b). Compared to basic conditions (98 [38–143] cm), the length was significantly shorter during the cognitive (89 [29–199] cm, *p* = 0.001, r = −0.532) and combined tasks (85 [35–137] cm, *p* < 0.001, r = −0.817), both with large effect sizes. The combined task also showed a significantly shorter stride length than the motor task (101 [47–193] cm, *p* < 0.001, r = −0.778), with a large effect size, and the cognitive task (*p* < 0.001, r = −0.579), with a large effect size. The cognitive task differed from the motor task (*p* = 0.013, r = −0.400) with a moderate effect size. No significant differences were observed between the motor and basic conditions (*p* = 0.994).

Foot clearance differed significantly across conditions (*p* = 0.003, W = 0.120; Figure 2c). Compared to basic conditions (9.5 [5.5–15.5] cm), clearance was significantly lower during the cognitive (9.0 [4.5–15.0] cm, *p* = 0.001, r = −0.516) and combined tasks (9.0 [5.0–15.0] cm, *p* = 0.001, r = −0.519), both with large effect sizes. The motor task (9.5 [4.0–15.0] cm) showed significantly different clearance compared to the basic condition (*p* = 0.036, r = −0.335), with a moderate effect size, and the combined task (*p* = 0.044, r = −0.323), with moderate effect size. No significant differences were observed between the combined and cognitive tasks (*p* = 0.771) or between the cognitive and motor tasks (*p* = 0.079).

The gait cycle duration differed significantly across conditions (*p* < 0.001, W = 0.312; Figure 3a). Compared to basic conditions (1.3 [0.90–4.55] s), the duration was significantly longer during both the cognitive (1.48 [1.00–4.20] s, *p* = 0.001, r = −0.533) and combined tasks (1.5 [1.00–3.10] s, *p* = 0.001, r = −0.532), both with large effect sizes. The combined task also showed a longer duration than the motor task (1.44 [0.90–5.60] s, *p* = 0.004, r = −0.452), with a moderate effect size, and the same was true for the cognitive versus motor task comparison (*p* = 0.005, r = −0.439), with a moderate effect size. No significant differences were observed between the motor and basic conditions (*p* = 0.816) or between the combined and cognitive conditions (*p* = 0.448).

The stance phase differed significantly across the conditions (*p* < 0.001, W = 0.295; Figure 3b). Compared to basic conditions (66.45 [61.80–90.75]%), the stance phase was significantly longer during both the cognitive (67.68 [61.10–89.45]%, *p* = 0.007, r = −0.428) and combined tasks (68.15 [59.00–88.85]%, *p* < 0.001, r = −0.665), showing moderate and large effect sizes, respectively. The combined task also demonstrated a significantly longer stance phase than both the motor task (66.35 [59.35–88.30]%, *p* < 0.001, r = −0.648), with a large effect size, and the cognitive task (*p* = 0.01, r = −0.407), with a moderate effect size. No significant difference was observed between the motor and basic conditions (*p* = 0.851).

The single-support phase differed significantly across conditions (*p* < 0.001, W = 0.236; Figure 3c). Compared to basic conditions (33.90 [8.45–38.25]%), the single-support phase was significantly reduced during the cognitive (32.52 [13.05–37.70] %, *p* = 0.006, r = −0.435) and combined tasks (32.03 [10.40–40.90]%, *p* = 0.001, r = −0.543), showing moderate and large effect sizes, respectively. The motor task (33.55 [8.65–41.15]%) also showed a significantly shorter single-support phase than the basic conditions (*p* = 0.007, r = −0.425), with a moderate effect size. The combined task demonstrated further reductions compared to both the motor (*p* = 0.002, r = −0.493) and cognitive tasks (*p* = 0.009, r = −0.410), both with moderate effect sizes. No significant difference was observed between the cognitive and motor tasks (*p* = 0.111).

The double-support phase differed significantly across the conditions (*p* < 0.001, W = 0.316; Figure 3d). Compared to basic conditions (32.5 [24.0–83.8]%), the double-support phase was significantly longer during both the cognitive (35.6 [24.2–77.2]%, *p* = 0.005, r = −0.442) and combined tasks (36.2 [18.1–81.6]%, *p* < 0.001, r = −0.659), showing moderate and large effect sizes, respectively. The combined task also demonstrated a significantly longer double-support phase than both the motor task (32.6 [18.2–82.9]%, *p* < 0.001, r = −0.585), with a large effect size, and the cognitive task (*p* = 0.004, r = −0.455), with a moderate effect size. No significant difference was observed between the motor and basic conditions (*p* = 0.323).

### 3.2. Kinematic Parameters

**Figure 4:**
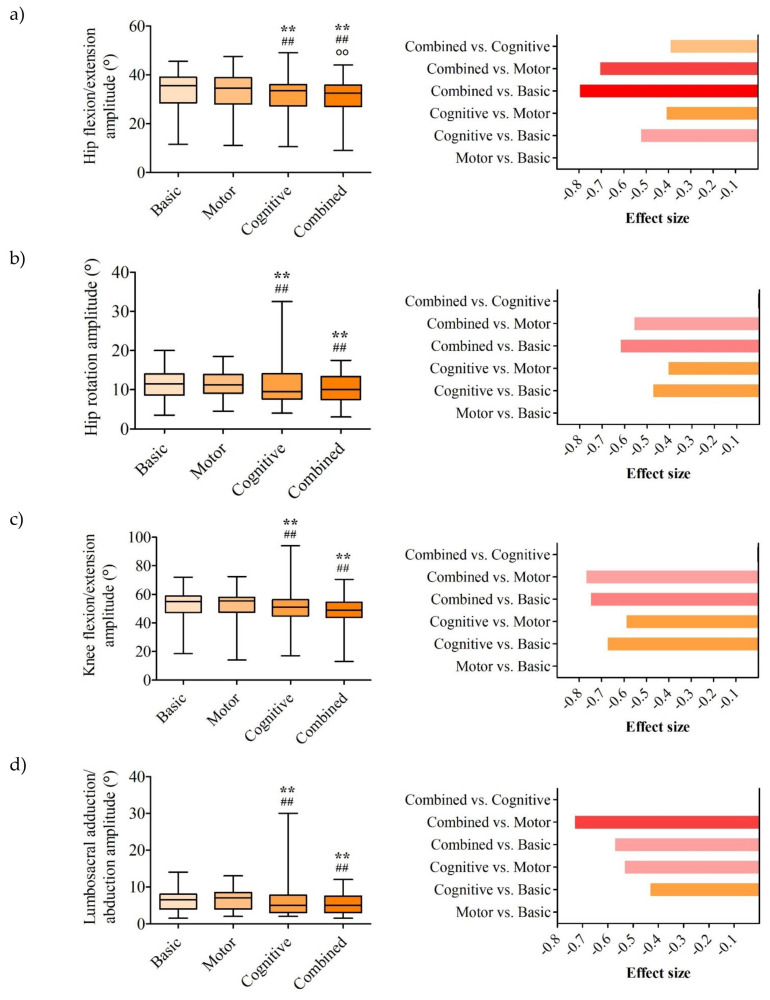
Kinematic parameter results—(**a**) hip flexion/extension amplitude; (**b**) hip rotation amplitude; (**c**) knee flexion/extension amplitude and (**d**) lumbosacral adduction/abduction amplitude. The left panel displays the median, interquartile range, minimum, and maximum values, along with statistically significant differences. The right panel shows the rank-biserial correlation coefficients for pairwise comparisons of parameters with statistically significant differences. *p* < 0.05 vs. basic condition; ** *p* < 0.01 vs. basic condition; ## *p* < 0.01 vs. during the motor activity; ○○ *p* < 0.01 vs. during the cognitive activity.

The hip flexion/extension amplitude differed significantly across the conditions (*p* < 0.001, W = 0.340; Figure 4a). Compared to the basic task (35.5 [11.5–45.5]°), the amplitude was significantly reduced in both the cognitive (33.5 [10.5–49.0]°, *p* = 0.002, r = −0.526) and combined tasks (32.25 [9.0–44.0]°, *p* < 0.001, r = −0.801), showing large effect sizes. The combined task also demonstrated a significantly lower amplitude than both the motor task (34 [11.0–47.5]°, *p* < 0.001, r = −0.710), with a large effect size, and the cognitive task (*p* = 0.018, r = −0.395), with moderate effect size. The cognitive task showed a reduced amplitude compared to the motor task (*p* = 0.013, r = −0.412), with a moderate effect size. No significant difference was observed between the motor and basic conditions (*p* = 0.104).

The hip rotation amplitude differed significantly across conditions (*p* < 0.001, W = 0.194; Figure 4b). Compared to basic conditions (11.5 [3.5–20.0]°), the amplitude was significantly reduced in both the cognitive (9.5 [4.0–32.5]°, *p* = 0.004, r = −0.476) and combined tasks (10.0 [3.0–17.5]°, *p* < 0.001, r = −0.622), showing moderate and large effect sizes, respectively. The combined task also showed a significantly lower amplitude than the motor task (11.25 [4.5–18.5]°, *p* = 0.001, r = −0.562), with a large effect size. No significant differences were observed between the motor and basic conditions (*p* = 0.729) or between the combined and cognitive tasks (*p* = 0.482).

There was no statistically significant difference in hip abduction/adduction amplitude across the four conditions (*p* = 0.098).

The knee flexion/extension amplitude differed significantly across conditions (*p* < 0.001, W = 0.466; Figure 4c). Compared to the basic task (55.0 [18.5–72.0]°), the amplitude was significantly reduced in both the cognitive (51.0 [17.0–94.0]°, *p* < 0.001, r = −0.677) and combined tasks (49.0 [13.0–70.5]°, *p* < 0.001, r = −0.752), both with large effect sizes. The combined task also showed a significantly lower amplitude than the motor task (55.0 [14.0–72.5]°, *p* < 0.001, r = −0.773), with a large effect size. The cognitive task demonstrated a reduced amplitude compared to the motor task (*p* < 0.001, r = −0.593), with a large effect size. No significant differences were observed between the combined and cognitive tasks (*p* = 0.128) or between the motor and basic tasks (*p* = 0.453).

The lumbosacral (pelvic) adduction/abduction amplitude differed significantly across conditions (*p* < 0.001, W = 0.344; Figure 4d). Compared to basic conditions (6.5 [1.5–14.0]°), the amplitude was significantly reduced in both the cognitive (5.0 [2.0–30.0]°, *p* = 0.008, r = −0.435) and combined tasks (5.0 [1.5–12.0]°, *p* < 0.001, r = −0.574), showing moderate and large effect sizes, respectively. The combined task also demonstrated a significantly lower amplitude than the motor task (7.0 [2.0–13.0]°, *p* < 0.001, r = −0.734), with a large effect size. The cognitive task showed a reduced amplitude compared to the motor task (*p* < 0.001, r = −0.536), with a large effect size. No significant difference was observed between the cognitive and combined conditions (*p* = 0.065).

No statistically significant difference was not found between the lumbosacral flexion/extension amplitude (*p* = 0.242) and the lumbosacral rotation amplitude (*p* = 0.173) across the four conditions.

## 4. Discussion

The current study examined spatiotemporal and kinematic gait parameters in healthy elderly participants using an IMU-based gait analysis system during cognitive–motor dual-task conditions. The analysis focused on comparing gait alterations between different dual-task paradigms to assess their relative impacts. Dual-task conditions significantly impaired gait parameters, with combined cognitive–motor tasks showing the strongest effects: reduced speed (*p* < 0.001), shorter strides (*p* < 0.001), and longer gait cycle durations (*p* < 0.001), with large effect sizes (r = −0.48 to −0.82). Kinematic parameters like hip mobility also decreased during dual tasks (flexion *p* < 0.001; rotation *p* < 0.001).

Cognitive tasks, notably, led to a prolonged gait cycle duration, impacting step rhythm more than motor tasks compared to basic conditions, with large effect sizes (r = −0.698, r = −0.533). Similarly, the combined task exhibited these effects, suggesting that cognitive demands significantly influence gait dynamics. Supporting these findings, Beauchet et al. [22] reported that older adults exhibited prolonged walking cycle durations during arithmetic tasks but not during verbal fluency tasks. This indicates that gait speed during dual tasks might be influenced by the type of cognitive task, reinforcing the notion that specific cognitive demands can more profoundly disrupt gait patterns. Additionally, increased gait cycle times were observed in similar studies when elderly individuals performed a verbal fluency task while walking [23] or engaged in the serial subtraction of seven from a three-digit number [24]. These studies collectively highlight how different cognitive tasks affect gait characteristics, with certain tasks inducing prolonged cycle durations.

The analysis demonstrated that cognitive and combined dual-task conditions significantly reduced foot clearance compared to baseline (*p* = 0.001 for both), with large effect sizes (r = −0.516 to −0.519), whereas the motor task alone showed only a moderate reduction (*p* = 0.036, r = −0.335), indicating that cognitive demands have a substantially greater impact on gait adaptation than motor interference [25].

Gait speed suffered pronounced reductions during cognitive and combined tasks, revealing significant walking inefficiency. Stride length decreased with cognitive elements, especially in the combined task versus the basic (r = −0.817) and cognitive tasks (r = −0.579), suggesting adaptive movement strategies. Additionally, combining walking with a mental tracking task through dual-tasking impacted spatiotemporal gait parameters, resulting in shorter stride lengths among the elderly individuals during these dual tasks [26]. Negative effects of arithmetic tasks on gait were noted, which align with previous research [27] indicating that older adults may prioritize cognitive tasks over walking, thereby significantly impacting their gait performance.

Significant changes were observed in the stance phase and single-support phase across the different conditions. The stance phase was significantly longer during combined tasks compared to both the basic and motor tasks, with large effect sizes (r = −0.665, r = −0.648). The single-support phase was noticeably shorter during combined tasks relative to the basic conditions, with a large effect size (r = −0.543) [28,29].

The double-support phase duration also changed significantly under cognitive demands, particularly in the combined task compared to both the basic and motor tasks (r = −0.659, r = −0.585), indicating alterations in weight distribution and potential instability requiring compensatory mechanisms. This prolonged double-support phase is linked to an increased gait cycle duration [30], which may reflect a compensatory strategy to decrease attentional demands during the swing phase, thereby reducing the risk of balance loss [31].

It can be concluded that older adults exhibit unique spatiotemporal gait patterns characterized by slower speeds, reduced cadences, shortened stride lengths, and prolonged single- and double-support times when engaged in dual tasks and that these observed patterns reinforce existing evidence [32].

The research also highlighted significant effects of task complexity on joint range of motion, particularly in the hips and knees. Hip flexion and extension were notably reduced during cognitive tasks (33.5°) compared to basic (35.5°) and motor tasks (34°), indicating that mental tasks hamper physical performance. The hip rotation amplitude showed significant differences as well, being notably lower during cognitive tasks (9.5°) and combined tasks (10.0°) compared to basic conditions (11.5°), with large effect sizes observed for combined versus basic (r = −0.622) and motor tasks (r = −0.562). Hip abduction and adduction showed no significant differences (*p* = 0.098), suggesting stability under cognitive load.

Knee flexion and extension decreased during cognitive tasks (51.0°) versus basic (55.0°) and motor tasks (55.0°), with the combined task showing even lower amplitudes (49.0°) compared to the basic task. In the lumbosacral/pelvic region, the adduction and abduction amplitudes of pelvis movement during the cognitive tasks were significantly lower (5.0°) compared to the basic (6.5°, *p* = 0.008) and motor tasks (7.0°, *p* < 0.001). Flexion, extension, and rotation in this area showed no statistical differences.

Prior research employing kinematic parameters has shown superior discriminative capacity for detecting balance impairments compared to spatiotemporal measures. Studies indicate that dual-task paradigms elicit subtle yet clinically meaningful kinematic deviations from single-task performance, with critical implications for assessing functional mobility [33]. These findings underscore the link between task complexity and movement amplitudes, with significant implications for rehabilitation and functional movement assessments, especially under mental load. Contrary to relying only on spatiotemporal data, some findings highlight that the kinematic analysis of segments of the lower extremities provides superior detection of gait abnormalities during dual-task walking [34].

It is important to note that individual differences in cognitive reserve are shaped by factors such as educational attainment and occupational background [35,36], which can, in turn, meaningfully influence dual-task performance. These background characteristics could affect both the cognitive demands perceived during tasks and the compensatory strategies employed while walking. For example, individuals with higher educational levels or cognitively demanding professional histories may demonstrate greater adaptability under dual-task conditions, potentially masking or mitigating observable gait disruptions. Although such variables were not collected in the present study, their potential influence should be considered when interpreting inter-individual variability in dual-task gait outcomes. Future investigations would benefit from incorporating these background factors to improve the precision and reproducibility of findings related to cognitive–motor interference.

While this research offers important insights into the effects of cognitive tasks on gait dynamics and joint range of motion, several limitations should be recognized. Firstly, the relatively small sample size of participants and the group sex imbalance may not adequately represent the broader population, potentially limiting the external validity of the results. The present study was designed as an exploratory investigation into the effects of different dual-task conditions on gait parameters in older adults. As such, no a priori power analysis was conducted. Instead, the sample size (n = 41) was determined based on feasibility constraints (availability of eligible and willing participants) and by referencing prior studies with a similar methodology and objectives, which included comparable or smaller samples. Nevertheless, the observed effect sizes in our results (many in the large range, e.g., r > 0.5) suggest that this study was adequately powered to detect meaningful differences across conditions.

A detailed assessment of participants’ medical history, comorbidities, and baseline cognitive and motor function was not performed, nor were standardized assessments of participants’ functional status, daily activity levels, or mobility independence (e.g., ADL, IADL, TUG, or SPPB) conducted. This limitation affects the clinical characterization of the sample and the contextualization of gait performance findings. Additionally, the study specifically examined certain cognitive tasks, such as arithmetic tasks, and did not consider a wider variety of cognitive demands, which could influence gait in different ways. Furthermore, the reliance on laboratory-based measurements may not fully reflect real-world walking behaviors, as various environmental factors can significantly impact gait performance. The cross-sectional design of the study restricts our ability to establish causal relationships between cognitive tasks and changes in gait, underscoring the need for longitudinal studies to explore these dynamics over time. Finally, certain participant characteristics such as handedness and limb dominance were not recorded in the present study. These variables may influence gait asymmetries and should be considered in future research to improve the interpretability and generalizability of findings. Additionally, as mentioned above, the absence of data on participants’ education and occupational background may have introduced variability that was unaccounted for, given their established relevance for cognitive reserve and dual-task performance [35,36].

Addressing these limitations in future research could enhance our understanding of how cognitive demands affect gait and inform better interventions for older adults.

## 5. Conclusions

The aging process is associated with a natural decline in cognitive resources, which manifests through measurable deteriorations in motor performance. As demonstrated in this study and supported by the existing literature, these cognitive declines contribute not only to reduced gait performance and elevated fall risk among older adults but also to specific alterations in dual-task gait parameters—a phenomenon that may serve as an early indicator of cognitive impairment. The observed kinematic and spatiotemporal changes highlight the profound interdependence between cognitive function and locomotor control. Notably, cognitive tasks act as a significant constraint on walking capabilities, inducing quantifiable adaptations in gait patterns. Participants frequently adopt compensatory strategies, such as slower velocities and increased postural adjustments, to mitigate stability challenges under dual-task conditions. These behavioral adaptations, coupled with the large effect sizes observed in gait measures, underscore the substantial impact of cognitive load on mobility. The consistency of these findings across studies suggests that dual-task gait analysis may offer clinical utility for the early detection of cognitive–motor deficits. Future research should explore the longitudinal relationships between dual-task gait decline and cognitive impairment progression, as well as the potential for targeted interventions to improve dual-task performance in aging populations. The integration of instrumented assessments (e.g., inertial sensors) into clinical practice could enhance the sensitivity of functional evaluations, moving beyond traditional single-task paradigms to better reflect real-world mobility challenges.

## Figures and Tables

**Table 1:** Descriptive characteristics of the participants.

	Male (n = 11)	Female (n = 30)	Total (N = 41)
Age (years)	68.7	71.9	71.1
Height (m)	1.83	1.62	1.67
Weight (kg)	81.5	71.5	73.9
BMI (kg/m^2^)	24.4	27.5	26.7

## Data Availability

Raw data will be made fully available upon reasonable request to the corresponding author.

## Abbreviations

The following abbreviations are used in this manuscript:

SD	Standard Deviation
ROM	Range of Motion
CoM	Center of Mass
IMU	Inertial Measurement Unit
